# Menopausal hormone use and ovarian cancer risk: individual participant meta-analysis of 52 epidemiological studies

**DOI:** 10.1016/S0140-6736(14)61687-1

**Published:** 2015-05-09

**Authors:** Collaborative Group on Epidemiological Studies of Ovarian Cancer

## Abstract

**Background:**

Half the epidemiological studies with information about menopausal hormone therapy and ovarian cancer risk remain unpublished, and some retrospective studies could have been biased by selective participation or recall. We aimed to assess with minimal bias the effects of hormone therapy on ovarian cancer risk.

**Methods:**

Individual participant datasets from 52 epidemiological studies were analysed centrally. The principal analyses involved the prospective studies (with last hormone therapy use extrapolated forwards for up to 4 years). Sensitivity analyses included the retrospective studies. Adjusted Poisson regressions yielded relative risks (RRs) versus never-use.

**Findings:**

During prospective follow-up, 12 110 postmenopausal women, 55% (6601) of whom had used hormone therapy, developed ovarian cancer. Among women last recorded as current users, risk was increased even with <5 years of use (RR 1·43, 95% CI 1·31–1·56; p<0·0001). Combining current-or-recent use (any duration, but stopped <5 years before diagnosis) resulted in an RR of 1·37 (95% CI 1·29–1·46; p<0·0001); this risk was similar in European and American prospective studies and for oestrogen-only and oestrogen-progestagen preparations, but differed across the four main tumour types (heterogeneity p<0·0001), being definitely increased only for the two most common types, serous (RR 1·53, 95% CI 1·40–1·66; p<0·0001) and endometrioid (1·42, 1·20–1·67; p<0·0001). Risk declined the longer ago use had ceased, although about 10 years after stopping long-duration hormone therapy use there was still an excess of serous or endometrioid tumours (RR 1·25, 95% CI 1·07–1·46, p=0·005).

**Interpretation:**

The increased risk may well be largely or wholly causal; if it is, women who use hormone therapy for 5 years from around age 50 years have about one extra ovarian cancer per 1000 users and, if its prognosis is typical, about one extra ovarian cancer death per 1700 users.

**Funding:**

Medical Research Council, Cancer Research UK.

## Introduction

**Figure 1:**
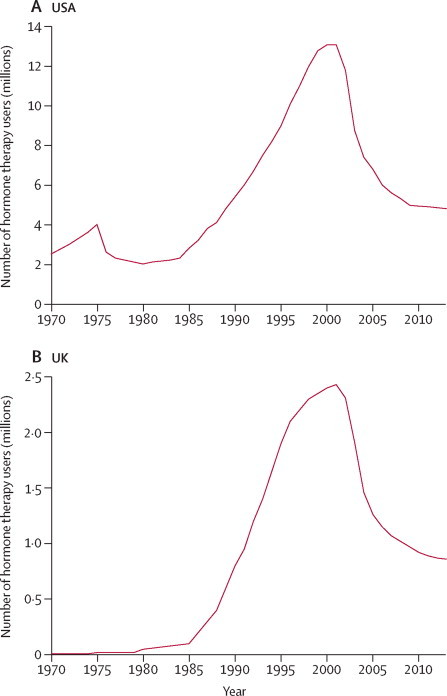
Trends in hormone therapy use in the USA and the UK since 1970 For source of data, see appendix p 4.

Use of hormone therapy for the menopause increased rapidly during the 1990s, then halved abruptly in the early 2000s after publication of the Women's Health Initiative, a large randomised trial,^1^ but has stabilised during the 2010s with about 6 million users in the USA and UK alone (figure 1, appendix p 4).

Current hormone therapy guidelines vary in what is said about ovarian cancer. The European drug regulatory guidelines^2^ do not mention the disease, nor does the US Food and Drug Administration statement^3^ (based just on the Women's Health Initiative, which recorded few ovarian cancers). UK drug regulatory guidelines^4^ state that ovarian cancer might be increased by long-term use, but were dominated by findings from one large study;^5^ new UK guidelines are being developed. The most recent WHO review was completed before results from most large studies were published, so merely concluded that there was insufficient evidence about any ovarian cancer risk.^6^ Recently, some non-governmental reviews have argued that a few years of hormone therapy use starting before the age of 60 years should cause no material harm.^7^ Most individual studies have, however, been too small to assess reliably any risks associated with use for only a few years (which is nowadays the usual pattern), so a systematic review of the worldwide epidemiological evidence is needed.

Reliable epidemiological assessment of any association of hormone therapy use with ovarian cancer requires large numbers and careful control of all potential sources of appreciable bias, and reviews just of the published evidence cannot provide this. For, although many studies of ovarian cancer collected some information about hormone therapy use, some were focused chiefly on other issues. Hence, published data about hormone therapy use are available for only about half the studies of ovarian cancer that have relevant data (appendix pp 5–9). Moreover, in some of the studies with retrospective designs, hormone therapy users might have been more willing than non-users to participate as controls, or there might have been differential recall of hormone therapy use between women already diagnosed with ovarian cancer and unaffected women. The Collaborative Group on Epidemiological Studies of Ovarian Cancer was established in 1998 to bring together and analyse centrally individual participant data from all epidemiological studies of ovarian cancer, assessing the risks associated with hormonal and other factors. To evaluate with minimal bias the association of ovarian cancer with just a few years of hormone therapy use, or with past use, the principal analyses review detailed data from those prospective studies with information about both duration and recency of hormone therapy use. Sensitivity analyses review the evidence from all studies, prospective or retrospective.

## Methods

### Identification of studies and collection of data

Since 1998, epidemiological studies, published and unpublished, have been sought regularly by computer-aided literature searches, manual searches of review articles, written communications, and discussions at scientific meetings (see appendix p 5 for search strategy). Eligible studies are those with information on hormone therapy use, parity, oophorectomy and hysterectomy, and, if completed after 2006, at least 200 cases of ovarian cancer. By January, 2013, 58 such studies were identified and principal investigators from each eligible study had been invited to collaborate. Datasets from 52 studies are included in these analyses and publications from three others are reviewed (appendix pp 5–10).

Cases are postmenopausal women with malignant, or borderline-malignant, epithelial or non-epithelial ovarian cancer; controls are postmenopausal women without ovarian cancer or previous oophorectomy.

In prospective studies, up to four randomly selected matched controls per case were selected. Individual data from 51 of the 52 studies were analysed centrally as case-control comparisons; because of data protection laws, individual data from the Danish Sex Hormone Register Study^8^ could not be exported, so its investigators provided detailed tabular results to combine with those of other studies (appendix p 6).

Information was sought for each woman on sociodemographic, reproductive, and other factors, including hormone therapy use before cancer diagnosis for cases and to an equivalent time for controls. Postmenopausal was defined as having reached natural menopause or age 55 years (because >90% of women have a natural menopause before that age^9^). Hysterectomy can mask natural menopause, so women younger than 55 years with a hysterectomy were excluded. Information sought about hormone therapy included ever-use, current use, age at first and last use, total duration of use, and constituents of each preparation. The hormone therapy preparation last used was classified as oestrogen-only or oestrogen-progestagen (or other/unknown formulation; appendix p 6).

Tumour histology was classified as fully malignant or borderline-malignant, and as epithelial or not. Epithelial tumours were further subdivided into the four most common types: serous, endometrioid, mucinous, or clear-cell (or mixed/other; appendix p 6). When appropriate, the International Classification of Diseases for Oncology^10^ was used.

Full details about information sources, search strategy, data collection, and definitions are provided in the appendix.

### Statistical analyses and presentation of results

A protocol was circulated to collaborators and preliminary results were discussed at a meeting of investigators in July, 2011. Poisson logistic regression comparing particular groups of hormone therapy users with never-users yielded odds ratios, described here as relative risks (RRs). When more than two groups were compared (eg, current-or-recent users, long-term ex-users, and never-users), the variance of the log risk was estimated for each group (appendix p 6).^11^ These group-specific variances were used to calculate group-specific CIs, facilitating valid comparisons between any two or more groups, whether or not one of them was designated as the baseline group.

The principal analyses include the prospective studies only, to avoid any possible biases associated with differential participation or recall in retrospective studies, but throughout the main report sensitivity analyses are given that include both the prospective and the retrospective studies. Results for the retrospective studies only are given in the appendix (pp 17, 19, 20), and heterogeneity tests were done to compare results from prospective and retrospective studies.

Because women can change their use of hormone therapy over time, follow-up in prospective studies was censored 4 years after hormone therapy use was last recorded (sensitivity analyses explored other cutoffs); duration and recency of use were estimated as if the last recorded use had continued (ie, duration of use in those who were current users when last asked increased by 1 year for each year of follow-up, as did time since last use of hormone therapy in ex-users). Hence, if in a prospective study of hormone therapy use the information last recorded before diagnosis is correct, then in analyses that combine current users with recent ex-users (ie, women who stopped <5 years before diagnosis), the current-or-recent users would include no misclassified women. Likewise, the never-users are contaminated with few hormone therapy users: only those who started in the interval of less than 4 years before diagnosis, which would be so uncommon (appendix p 6) as to dilute only slightly any real effects of hormone therapy use on risk.

To ensure that women in one study were compared directly only with similar women in that same study, all analyses were stratified by study, centre within study, age (5-year age groups up to 85–89 years), and body-mass index (<25, 25–29, or ≥30 kg/m^2^), and were adjusted for parity (0, 1–2, or ≥3), past use of oral contraceptives (never, <5 years of use, or ≥5 years of use), and age at menopause (natural menopause before age 50 years, natural menopause after age 50 years, or previous hysterectomy). Unknowns for each variable were assigned to separate strata. Sensitivity analyses investigated additional adjustment for eight other potential confounding factors. Standard χ^2^ tests for heterogeneity were used.

Results were weighted by the amount of statistical information in each stratum (inverse of the variance of log RR) and are presented as squares and lines, representing RRs and CIs (or, where appropriate, group-specific CIs). Study-specific results give 99% CIs (to allow for multiple testing), but most other results in the figures and all results in the text have 95% CIs. To illustrate the correspondence between relative and absolute risks in hormone therapy users, absolute risks were estimated from ovarian cancer incidence rates in England (appendix p 11).^12^ Analyses were done with STATA 13.

### Role of the funding sources

The study funders had no role in study design, data collection, analysis or interpretation, report preparation, or the decision to publish. The analysis and writing committee had full access to all the data and had final responsibility for the decision to submit for publication.

## Results

Overall information was provided for 21 488 postmenopausal women with ovarian cancer (cases) from 52 studies (17 prospective and 35 retrospective; appendix pp 7–10). The prospective studies contributed more than half of the cases (12 110), with mean diagnosis year 2001 (SD 6), 55% (6601) of whom had used hormone therapy, with median duration 6 years (IQR 2–10). By contrast, in the retrospective studies only 29% (2702) of the women had used hormone therapy, with median duration 4 years (IQR 1–10), and the mean diagnosis year was 1992 (SD 8), well before peak hormone therapy use (figure 1).

Ovarian cancer risk was significantly greater in ever-users than in never-users of hormone therapy (RR 1·20, 95% CI 1·15–1·26, p<0·0001 for prospective studies; 1·14, 1·10–1·19, p<0·0001 for all studies combined; every study-specific result is provided in appendix p 12). Subsequent analyses were restricted to women with information both on duration of use and on time since last use of hormone therapy; this exclusion of studies without information on duration of use or time since last use slightly increased these RRs (appendix p 13).

**Figure 2:**
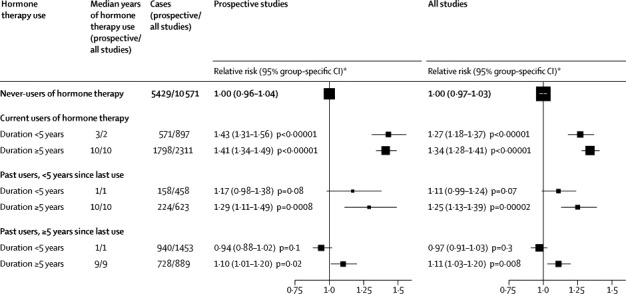
Relative risk of ovarian cancer by duration of use in current and past users of hormone therapy *Risk relative to never-users of hormone therapy, stratified by age at diagnosis, study, and body-mass index, and adjusted for age at menopause, hysterectomy, oral contraceptive use, and parity. p values are two-sided and include the effects of the group-specific variance in never-users.

Risk was strongly related to recency of use (figure 2). In prospective studies, risk was greatest in women who when last asked had been current users (RR 1·41, 95% CI 1·32–1·50; p<0·0001). Among them, risk was substantial even in those who, at diagnosis, had less than 5 years (median duration 3 years) of hormone therapy use (RR 1·43, 95% CI 1·31–1·56; p<0·0001).

Risk was, however, also significantly increased in women who had been recent ex-users and would at the time of diagnosis have still have been within 5 years of last use (RR 1·23, 95% CI 1·09–1·37; p=0·0006 in prospective studies). Risk decreased the longer ago hormone therapy had last been used, although women who had used hormone therapy for at least 5 years (median duration 9 years) and then stopped were still at significantly increased risk more than 5 years (median time since last use 10 years) later (RR 1·10, 95% CI 1·01–1·20; p=0·02). For prospective and retrospective studies combined, the risks were similar to those in prospective studies alone, except that the risks in current users seemed to be somewhat smaller (figure 2, appendix p 17).

**Figure 3:**
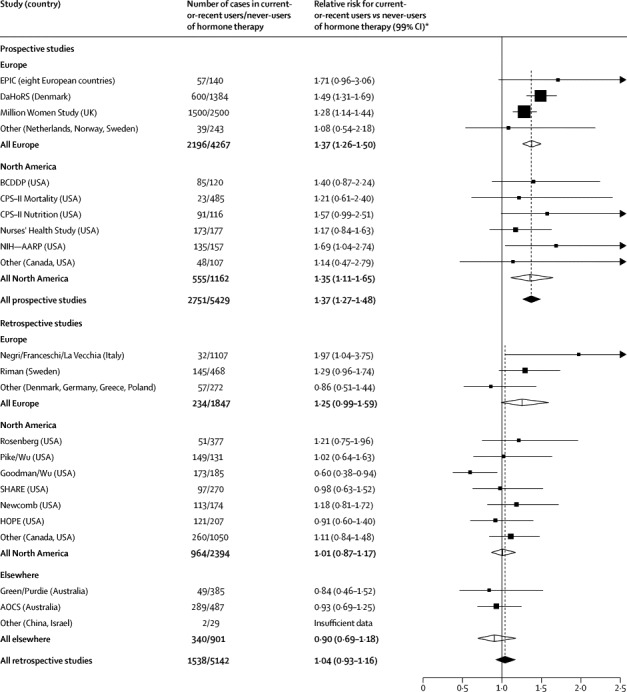
Study-specific results for the relative risk of ovarian cancer for current-or-recent users versus never-users of hormone therapy For study-specific details and references, see appendix pp 7–10. Dotted lines represent totals for all prospective studies and, separately, for all retrospective studies. Study-specific results are arranged by study design and region; results are given for individual studies with the most statistical information (ie, with variance of log relative risk <0·03). Results for the remaining studies are grouped together here (and given separately for every study in appendix p 14). In comparisons of relative risks in prospective versus retrospective studies, overall heterogeneity p<0·0001; for European studies, heterogeneity p=0·4; and for North American studies, heterogeneity p=0·002. In a comparison of relative risks in prospective studies, Europe versus North American heterogeneity p=0·9; for retrospective studies, Europe versus North American heterogeneity p=0·04. References provided in the appendix. *Risk relative to never-users of hormone therapy, stratified by age at diagnosis, study, and body-mass index, and adjusted for age at menopause, hysterectomy, oral contraceptive use, and parity.

In prospective studies the risk for current-or-recent hormone therapy use (ie, use within the past 5 years) was 1·37 (95% CI 1·27–1·48; figure 3). This RR did not differ significantly between European and North American studies (1·37 *vs* 1·35; heterogeneity p=0·9).

However, the RR for current-or-recent hormone therapy use did differ significantly between prospective and retrospective studies (1·37 *vs* 1·04; heterogeneity p<0·0001; figure 3, appendix p 14). This difference was due to the lack of apparent effect in the aggregated North American retrospective studies (figure 1, appendix pp 12–14). In these retrospective studies, however, the design might have made unbiased recruitment of controls difficult, and the average year of diagnosis for the cases was 1990, well before hormone therapy use had become common (figure 1).

Sensitivity analyses left the main findings in prospective studies largely unchanged (appendix p 18). For example, adjustment for eight additional factors (year of birth, ethnic origin, education, age at menarche, height, alcohol consumption, smoking, and family history of ovarian or breast cancer) altered the RRs in current-or-recent users by 0·02 or less (the main findings had already been stratified by study, age, and body-mass index, and adjusted for parity, use of hormonal contraceptives, age at menopause, and hysterectomy).

Furthermore, the main findings were robust against variation in follow-up duration. Censoring either earlier or later than year 4 (appendix p 18) made little difference to the results. Two prospective studies (in the UK^5^ and Denmark^8^) contributed the most statistical information, but the RR in all other prospective studies was much the same when these two were excluded. One prospective study^13^ used fatal ovarian cancer as the outcome, but again its findings were typical.

In current-or-recent users, ovarian cancer risk was significantly increased with use of both oestrogen-only and oestrogen-progestagen preparations, with little heterogeneity between the risks: RR 1·37 (95% CI 1·26–1·50) and 1·37 (1·26–1·48), respectively, in the prospective studies (heterogeneity p=0·9); and 1·32 (1·23–1·41) and 1·25 (1·16–1·34) in all studies (heterogeneity p=0·3; appendix p 17). Few women had used both these classes of hormone therapy, and within the two classes there was insufficient information to assess whether risk varied by hormone therapy formulation or mode of delivery (appendix p 6).

All but three studies provided tumour histology (appendix p 6). Of tumours with known histology, 98% (14 862 of 15 090) were epithelial. The RR in current-or-recent users versus never-users did not seem to differ between epithelial (RR 1·28, 95% CI 1·22–1·34) and non-epithelial (1·35, 0·90–2·02) tumours, although the confidence interval for non-epithelial tumours was wide. There were only 228 non-epithelial tumours, too few for further analysis.

**Figure 4:**
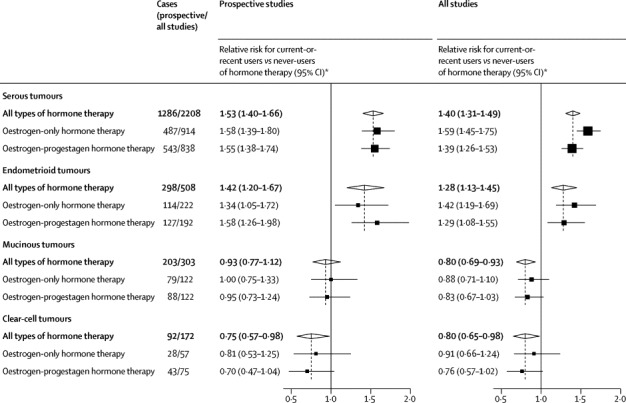
Relative risk of the four most common subtypes of ovarian cancer in current-or-recent users versus never-users of hormone therapy Numbers do not add to totals, because some hormone therapy users were classified as using other or unknown types of hormone therapy and some epithelial tumours are classified as mixed types, other type, or type not specified. *Risks relative to never-users of hormone therapy, stratified by age at diagnosis, study, and body-mass index, and adjusted for age at menopause, hysterectomy, oral contraceptive use, and parity.

Almost all epithelial tumours of known, unmixed histology were adenocarcinomas of four main tumour types which were, in decreasing order of frequency, serous (7406 cases), endometrioid (1749), mucinous (1434), and clear cell (766). These four epithelial tumour types had qualitatively different relationships with hormone therapy use, both in prospective studies alone and in all studies combined (figure 4; heterogeneity p<0·0001).

In prospective studies, risks in current-or-recent users were definitely increased only for the two most common tumour types, serous (RR 1·53, 95% CI 1·40–1·66, p<0·0001) and endometrioid (1·42, 1·20–1·67; p<0·0001). In the aggregate of these two types, the risk more than 5 years since last use for past users who had used hormone therapy for more than 5 years (RR 1·25, 95% CI 1·07–1·46; p=0·005) was more definite than in the aggregate of all types (1·10, 1·01–1·20, p=0·02). Risk might have been somewhat decreased for the least common type, clear-cell tumours (RR 0·75, 95% CI 0·57–0·98; p=0·04 before any allowance for multiple hypothesis testing), but this protective effect is not statistically definite, and in the aggregate of both of the less common types the risk reduction was not significant (RR 0·86, 0·74–1·01; p=0·07). Within each tumour type there was little difference between the RRs for oestrogen-only and oestrogen-progestagen preparations (figure 4), or for borderline and fully malignant tumours (appendix p 19).

Age at initiation of hormone therapy had little effect; the RRs in current-or-recent users were similarly elevated with hormone therapy use beginning before age 50 years (RR 1·35, 95% CI 1·24–1·47) and at age 50–59 years (1·31, 1·22–1·40), with little information about older ages (1·15, 0·93–1·43; appendix p 20). Likewise, the available evidence suggested no major heterogeneity across subgroups defined by smoking, body size, parity, past use of oral contraceptives, hysterectomy, or other characteristics (appendix pp 15–16).

Application of the RRs in the prospective studies to age-specific ovarian cancer incidence and death rates in England suggested that 5 years of hormone therapy use, starting at around 50 years of age, would result in about one additional ovarian cancer per 1000 users and one additional ovarian cancer death per 1700 users (appendix p 11); 10 years of hormone therapy use from around 50 years of age would result in about one additional ovarian cancer per 600 users and one additional ovarian cancer death per 800 users (table).

**Table:** Estimated excess incidence of ovarian cancer in England associated with 5 years and with 10 years of hormone therapy use, starting at age 50 years

	**5 year incidence of ovarian cancer per 1000 never-users of hormone therapy**	**Absolute 5 year excess incidence per 1000 users with 5 years of hormone therapy use**	**Absolute 5 year excess incidence per 1000 users with 10 years of hormone therapy use**
Age 50–54 years	1·2	0·52	0·52
Age 55–59 years	1·6	0·37	0·67
Age 60–64 years	2·1	0·10	0·61
Excess incidence	..	0·99 per 1000; 1 in 1000 users	1·80 per 1000; 1 in 600 users
Excess deaths	..	0·6 per 1000; 1 in 1700 users	1·2 per 1000; 1 in 800 users

Methods and sources of data are provided in appendix p 11.

## Discussion

This collaboration brought together and analysed centrally individual data from 52 epidemiological studies, in which about half the postmenopausal women with ovarian cancer had used hormone therapy. Ovarian cancer risk was significantly increased in current users, even in those with less than 5 years hormone therapy use. In ex-users, risks decreased the longer ago hormone therapy use had ceased, but risks during the first few years after stopping remained appreciable. Furthermore, about a decade after ceasing long-duration hormone therapy use, there still seemed to be a small excess risk.

In current-or-recent users (all of whom had used hormone therapy within the past 5 years), the RRs did not differ significantly between users of oestrogen-only and of oestrogen-progestagen preparations, or between women who had started hormone therapy before the age of 50 years or during their 50s. The RR did, however, vary substantially by tumour type, being increased only for the two most common histological types, serous and endometrioid tumours. In analyses restricted to these two types, the excess risk about a decade after ceasing long duration hormone therapy use became more definite.

An important strength of prospective studies is that recruitment takes place and information about hormone therapy use is recorded before women know whether they will develop ovarian cancer. The robustness of prospective data is demonstrated by the stability of the findings in various sensitivity analyses, and by the similarity of the findings in Europe and North America. Prospective studies provided more than half the statistical information, so results for all studies (prospective and retrospective combined) were broadly similar to those for the prospective studies alone.

When the retrospective studies were assessed in isolation, their aggregate findings differed from those of the prospective studies, however, perhaps because of biases in some retrospective studies. Many retrospective study results could have been somewhat biased by selective participation of hormone therapy users, and in all retrospective studies information about hormone therapy use was recorded after cancer diagnosis, so there might have been differential recall of hormone therapy use. Moreover, some retrospective datasets in this collaboration have yielded apparently discrepant findings on the association of ovarian cancer risk with smoking^14^ and with body-mass index.^15^

Almost all the worldwide evidence from eligible epidemiological studies was included in this meta-analysis. The three eligible studies that had published results but did not contribute data were all retrospective and North American, and had all reported increased risks of ovarian cancer associated with some aspect of hormone therapy use (appendix p 5). Had they been included, the findings in North American retrospective studies might not have been as different from those in other groups of studies.

As long as current users and recent ex-users are combined together as current-or-recent users, there are fewer potential sources of bias in prospective than in retrospective studies, and there is now for the first time sufficient evidence from prospective studies alone for statistically stable meta-analyses. Hence, it is now possible to base the main conclusions on prospective study results.

The overall relative risk for any type of ovarian cancer is the key public health outcome. There are, however, four main types of ovarian cancer, and in the prospective studies risk was definitely increased only for the two most common types, serous and endometrioid. Risk was possibly, although not definitely, decreased for the least common type, clear-cell tumours. Although tumour histology could have been classified in slightly different ways in different studies, any misclassification would tend to blur differences by tumour type, yet we noted distinctly heterogeneous RRs. This heterogeneity argues strongly for causality, because it implies that the hormone-therapy-associated risks were not due just to confounding and that different ovarian cancer types have differing causes. The reasons for this heterogeneity are unclear, partly because the sites of origin of the four main tumour types are uncertain.^16^ The dependence of risk on ovarian tumour type is quite different for other exposures; oral contraceptives decrease serous, endometrioid, and clear-cell but not mucinous tumours,^17^ whereas smoking decreases endometrioid and clear-cell but increases mucinous tumours.^14^

The findings that ovarian cancer risk is greatest in current users of hormone therapy, falls after use ceases, and varies by tumour type, strongly suggest a causal relationship—ie, that among otherwise similar women, use of hormone therapy increases the probability of developing the two most common types of ovarian cancer, and hence ovarian cancer as a whole. There are still some 6 million users of hormone therapy in the USA and the UK, in addition to a comparable number in other high-income countries (figure 1, appendix p 4). At present, the WHO, European, and US guidelines about hormone therapy do not mention ovarian cancer, and the UK guidelines (which are due to be revised) state only that risk may be increased with long-term use. The definite risk of ovarian cancer that is observed even with less than 5 years of use starting at around age 50 years is directly relevant to current patterns of hormone therapy use, and hence directly relevant to medical advice, personal choices, and the current efforts to revise UK and worldwide guidelines.
