# Supplementary material for: Menopausal hormone use and ovarian cancer risk: individual participant meta-analysis of 52 epidemiological studies
Source: Lancet. 2015 May 9;385(9980):1835–42. doi: 10.1016/S0140-6736(14)61687-1 (PMC4427760; doi:10.1016/S0140-6736(14)61687-1)
Supplement: Supplementary appendix [file mmc1.pdf]

THE LANCET

Supplementary appendix

This appendix formed part of the original submission and has been peer reviewed. We post it as supplied by the authors.

Supplement to: Collaborative Group on Epidemiological Studies of Ovarian Cancer. Menopausal hormone use and ovarian cancer risk: individual participant meta-analysis of 52 epidemiological studies. *Lancet* 2015; published online Feb 13. http://dx.doi.org/10.1016/S0140-6736(14)61687-1.

CONTENTS LIST: Click on any item to jump to it

Collaborators (page 3)

Patterns of use of HT over time (page 4)

Search strategy and eligibility criteria; studies not included (page 5)

Data collection, definitions and statistical methods (page 6)

Study-specific data and references (pages 7-10)

Estimated absolute risk of ovarian cancer in HT users (page 11)

Webfigures (pages 12-16)

Webfigure 1 Study-specific results for the relative risk of ovarian cancer in ever vs never users of HT (p12)

Webfigure 2 Study-specific results for the relative risk of ovarian cancer for ever versus never users of HT in women for whom there was also information on duration and time since last use of HT (p13)

Webfigure 3 Study-specific results for the relative risk of ovarian cancer for current-or-recent users (p14)

Webfigure 4 Relative risk of ovarian cancer in current-or-recent vs never users of HT, by subgroup; prospective studies only (p15).

Webfigure 5 Relative risk of ovarian cancer in current-or-recent versus never users of HT, by subgroup all studies (p16).

Webtables (pages 17-20)

Relative risk of ovarian cancer by duration and time since last HT use in retrospective studies (p17)

Sensitivity analyses in prospective studies (p18)

Relative risk of ovarian cancer in current-or-recent users by study design and tumour characteristics (p19)

Relative risk of ovarian cancer in current-or-recent users by study design and age at first use of HT (p20)

Analysis and writing committee: V Beral, K Gaitskell, C Hermon, K Moser, G Reeves (Oxford Cancer Epidemiology Unit) and R Peto (Oxford Clinical Trial Service Unit and Epidemiological Studies Unit [CTSU]).

Advisory committee: K Anderson, L Brinton, P Coogan, S Franceschi, S Gapstur, P Marchbanks, K Malone, P Newcomb, S Sadetzki, RM Tamimi, P Webb.

Collaborative Group on Epidemiological Studies of Ovarian Cancer: American Cancer Society, Atlanta, GA: SM Gapstur, AV Patel; Australian National University: E Banks; Aviano Cancer Center, Italy: L Dal Maso, R Talamini; Gertner Institute, Tel Hashomer, Israel: A Chetrit, G Hirsh-Yechezkel, F Lubin, S Sadetzki; Cancer Epidemiology Unit, Oxford, UK: V Beral, D Bull, B Cairns, B Crossley, K Gaitskell, A Goodill, J Green, C Hermon, T Key, K Moser, G Reeves; Cancer Council NSW, Australia: F Sitas; Clinical Trial Service Unit and Epidemiological Studies Unit (CTSU), Oxford, UK: R Collins, R Peto; Catalan Institute of Oncology, Barcelona, Spain: CA Gonzalez; Centers for Disease Control and Prevention, Atlanta, GA: N Lee, P Marchbanks, HW Ory, HB Peterson, PA Wingo; Chiang Mai University, Thailand: N Martin, S Silpisornkosol, C Theetranont; Chulalongkorn University, Bangkok, Thailand: B Boosiri, S Chutivongse, P Jimakorn, P Virutamasen, C Wongsrichanalai; Cedars-Sinai Medical Center, Los Angeles, CA: MT Goodman; Copenhagen University Hospital, Denmark: O Lidegaard, SK Kjaer, LS Morch; Danish Cancer Society Research Center, Copenhagen, Denmark: SK Kjaer, A Tjonneland; Colorado School of Public Health, Denver, CO: T Byers; Albert Einstein College of Medicine, Bronx, New York: T Rohan; Herlev University Hospital, Copenhagen, Denmark: B Mosgaard; Department of Public Health, Oxford, UK: M Vessey, D Yeates; Department of Social and Preventive Medicine, State University of New York, Buffalo, NY: JL Freudenheim; Geisel School of Medicine at Dartmouth, Hanover, NH: LJ Titus; German Cancer Research Center (DKFZ), Heidelberg, Germany: J Chang-Claude, R Kaaks; University of Minnesota School of Public Health, Minneapolis, MN: KE Anderson, D Lazovich, K Robien; Fred Hutchinson Cancer Research Center, University of Washington, Seattle, WA: J Hampton, PA Newcomb, MA Rossing, DB Thomas, NS Weiss; Hillerod Hospital, University of Copenhagen, Denmark: E Lokkegaard; Imperial College London, UK: E Riboli; INSERM U1018 and Paris South University, Institut Gustave-Roussy, Villejuif, France: F Clavel-Chapelon; Harvard Medical School, Cambridge, MA: D Cramer, SE Hankinson, RM Tamimi, SS Tworoger; International Agency for Research on Cancer, Lyon, France: S Franceschi; Istituto di Ricerche Farmacologiche Mario Negri, University of Milan, Italy: C La Vecchia, E Negri; Karolinska Institutet, Stockholm, Sweden: HO Adami, C Magnusson, T Riman, E Weiderpass, A Wolk; Maastricht University, Netherlands: LJ Schouten, PA van den Brandt; Mahidol University, Bangkok, Thailand: N Chantarakul, S Koetsawang, D Rachawat; Cancer Research and Prevention Institute, Florence, Italy: D Palli; National Cancer Institute, Bethesda, MD: A Black, LA Brinton, DM Freedman, P Hartge, AW Hsing, JV Lacey Jr, J Lissowska, RN Hoover, C Schairer; Cancer Epidemiology Research Group, National Health Laboratory Services, Medical Research Council, Johannesburg, S Africa: C Babb, M Urban; Norwegian Institute of Public Health, Oslo, Norway: S Graff-Iversen, R Selmer; QIMR Berghofer Medical Research Institute and University of Queensland: CJ Bain, AC Green, DM Purdie, V Siskind, PM Webb; Roswell Park Cancer Institute, Buffalo, NY: K Moysich, SE McCann; Royal College of General Practitioners' Oral Contraception Study, London, UK: P Hannaford, C Kay; School of Public Health, Curtin University, Perth, Australia: CW Binns, AH Lee, M Zhang; School of Public Health, University of Texas, Houston, TX: RB Ness; School of Public Health and Health Sciences, University of Massachusetts, Boston, MA: P Nasca; Slone Epidemiology Center, Boston University, MA: PF Coogan, JR Palmer, L Rosenberg; Stanford University, CA: A Whittemore; University of Athens Medical School, Athens, Greece: K Katsouyanni, A Trichopoulou, D Trichopoulos, A Tzonou; University of Chile, Santiago, Chile: A Dabancens, L Martinez, R Molina, O Salas; University of Hawaii, Honolulu, HA: G Lurie, ME Carney, LR Wilkens; University Hospital, Lund, Sweden: L Hartman, J Manjer, H Olsson; University Hospital of North Norway: M Kumle; University of Pennsylvania, Philadelphia, PA: JA Grisso, M Morgan, JE Wheeler; University of Pittsburgh, PA: RP Edwards, JL Kelley, F Modugno; University Medical Center, Utrecht, Netherlands: NC Onland-Moret, PHM Peeters; University of Southern California, Los Angeles, CA: J Casagrande, MC Pike, AH Wu; University of New South Wales, Australia: K Canfell; University of Toronto, Ontario, Canada: AB Miller; Arctic University of Tromso, Norway: IT Gram, E Lund; George Washington University, Washington, DC: L McGowan; Vanderbilt University, Nashville, TN: XO Shu, W Zheng; World Health Organization, Geneva, Switzerland: TMM Farley, S Holck, O Meirik; Yale School of Public Health, CT: HA Risch.

Patterns of use of HT over time

Over the last few decades the prevalence of HT use has varied considerably over time.† Detailed statistics on long term trends in HT use are available for the USA and the UK.

In the USA HT use began to increase in the early 1970s but declined in the late 1970s following reports of increased risks of endometrial cancer associated with use of oestrogen-only preparations. HT use began to increase again in the late 1980s, continued to increase during the 1990s and halved abruptly in the early 2000s (Figure A). Use stabilized during the 2010s, with an estimated 5 million users. The prevalence of HT use among controls from Canada in this meta-analysis appear broadly similar to those seen in the USA.

In the UK there was little use of HT until the late 1980s. HT use increased rapidly during the 1990s, halved abruptly in the early 2000s, and stabilized in the 2010s, with about 1 million users (Figure B).

In western and northern Europe and Australasia the patterns of HT use are broadly similar to those seen in the UK.

Estimated person-years of HT use since 1970

Assuming that the average duration of HT use was 5 years (as found for controls in this meta-analysis) it is estimated that have been about 600 million woman-years of HT use in high income countries since the 1970s, about half in North America and half in Europe and Australasia.

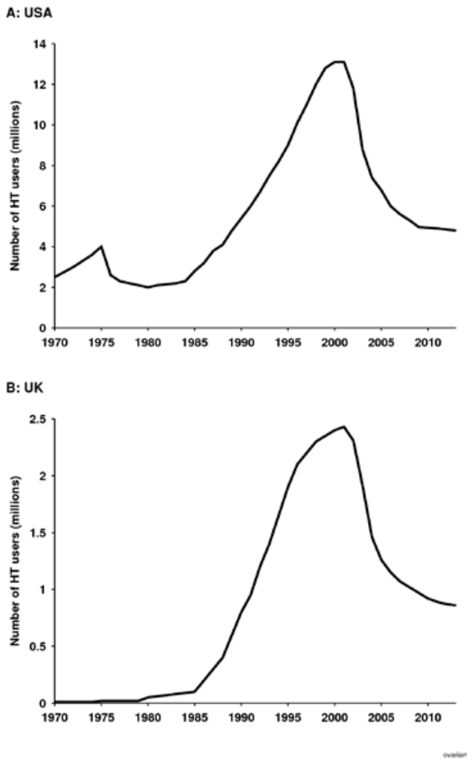

†Sources of data on HT use in different countries:

Kennedy DL et al. Noncontraceptive estrogens and progestins. Use patterns over time. *Obstet Gynecol* 1985;**65**:441-6.

Wysowski DK et al. Use of menopausal estrogens and medroxyprogesterone in the United States, 1982-1992. *Obstet Gynecol* 1995;**85**:6-10.

Townsend J. Hormone replacement therapy: Assessment of present use, costs and trends. *Br J Gen Pract* 1998; **48**: 955-8.

International Agency for Research on Cancer (IARC). Evaluation of Carcinogenic Risks to Humans, volume 72: Hormonal contraception and postmenopausal hormone therapy. IARC, Lyon, 1999.

Banks E et al. Use of hormonal therapy for the menopause in 9 European countries: results from the EPIC cohort. Pp 301-303 in: Riboli E, Lambert R (eds). IARC Scientific Publication No 156. IARC, Lyon, 2002.

Wysowski DK and Governale LA, Use of menopausal hormones in the United States, 1992 through June, 2003. *Pharmacoepidemiology and drug safety* 2005;**14**:171-76.

Watson J et al. Prescribing of hormone therapy for menopause, tibolone, and bisphosphonates in women in the UK between 1991 and 2005. *Eur J Clin Pharmacology* 2007;**63**:843-49.

Canfell K et al. Decrease in breast cancer incidence following a rapid fall in use of hormone replacement therapy in Australia. *Med J Aust* 2008;**188**:641-44.

Ettinger B et al. Evolution of post-menopausal hormone therapy between 2002 and 2009. *Menopause* 2012;**19** 610-15.

Steinkellner AR et al. A decade of postmenopausal hormone therapy prescribing in the United States. *Menopaus*e 2012;**19**:616-21.

NHS Prescription Cost Analysis, England. http://www.hscic.gov.uk/article/1165/Search-catalogue?q =title:Prescription+Cost+Analysis &area=&size=10&sort=RelevanceDesc (accessed 23 June 2014).

Search strategy and eligibility criteria

This collaboration began in 1998, and since then potentially eligible epidemiological studies have been sought regularly by searches of review articles and computer-aided literature searches in MEDLINE and PubMed, using combinations of the search terms 'ovarian cancer risk', 'ovary cancer risk', 'hormon*', 'HRT', 'HT', and 'menopause'. To be eligible for these analyses, studies needed to have collected individual data on women's use of hormonal therapies for the menopause and on their parity and past history of oophorectomy and of hysterectomy. Studies completed after 2006 needed to have included a total of at least 200 women with ovarian cancer (not all postmenopausal). Studies completed before then were eligible with fewer than 200 cases. Studies that had collected relevant data, but had not published on ovarian cancer risk in relation to use of HT, were sought by correspondence with colleagues, by discussions at collaborators meetings (in 2000, 2005 and 2011), and by electronic searches using additional terms 'cohort', 'prospective', 'women' and 'cancer risk'.

By January 2013, 58 eligible studies[1-58] had been identified and principal investigators from each had been invited to participate in the collaboration. Data from 52 of the eligible studies are included in these analyses.[1-52]

Studies not included in the analysis

Data from six eligible studies[53-58] were not included in these analyses data. Three[53-55] had not published on the relationship between HT use and ovarian cancer risk.

All three eligible studies that had published results[56-58] and could not contribute data to this analysis were retrospective studies done in North America. Mills et al[56] studied 256 women with ovarian cancer and the adjusted relative risk in ever vs never users of HT was reported to be 1.39, 95%CI 1.01-1.93. Moorman et al[57] studied 364 postmenopausal women and the adjusted relative risk in ever vs never users of HT was reported to be 1.2, 95% CI 0.8-1.6. Rossing et al[58] reported results for a subgroup of women with ovarian cancer who had either never used HT or had exclusively used either estrogen-only, continuous estrogen-progestin or sequential estrogen-progestin preparations; no estimate for ever versus never use of HT in all women was published.

For the two studies[56,57] that had presented data on ever versus never use of HT the combined relative risk was 1.3 (95%CI 1.0-1.5). No study published estimates of the overall relative risk of ovarian cancer in current or recent ex-users versus never users, but Moorman et al[57] reported relative risks of 1.1 (0.7-1.5) and 1.6 (0.9-2.8), respectively, for current use and for past users who had ceased in the previous 5 years (ie, 1.2 [0.9-1.7] in current-or-recent vs never users of HT)

Studies were ineligible if they had included cases that overlapped with those in other studies included in the collaboration. For example, cases in a retrospective study in Denmark (Glud E et al. Hormone therapy and the impact of estrogen intake on the risk of ovarian cancer. *Arch Int Med* 2004; 164: 2253-59) overlapped with cases in the larger prospective Danish Sex Hormone Register Study (DaHoRS).[45] Studies were also ineligible if no information on oophorectomy was available (eg, Koskela-Niska V et al. Effect of various forms of postmenopausal hormone therapy on the risk of ovarian cancer - a population-based case-control study from Finland. *Int J Cancer* 2013; 133: 1680-88).

Data collection and definitions

Individual participant data contributed by principal investigators were checked and collated centrally so that analyses could use definitions that were as similar as possible across studies. Apparent inconsistencies in the data were rectified, where possible, by correspondence with the investigators. After the records had been checked and corrected, investigators were sent summary tables and listings of the variables to be used in analyses for final confirmation.

Due to Danish data protection laws, data from one prospective study, the Danish Sex Hormone Register Study (DaHoRS)[45] could not transfer individual exposure data abroad, and could be accessed only by on-line submission to Statistics Denmark. Principal investigators adapted the Danish dataset according to the specifications for this meta-analysis and provided tabular results. Analyses were restricted to women aged 55 years and older and adjustment variables were age, past hysterectomy, and parity. The tabulated results were used to impute variance-covariance matrices of relative risk (Greenland S, Longnecker MP. Methods for trend estimation from summarized dose-response data, with applications to meta-analysis. *Am J Epidemiol* 1992; 135: 1301-09) and results were combined seamlessly with the results from the other 51 studies, weighted by the inverse of the estimated variance.

Definition of HT use

Information sought from principal investigators about every woman's use of HT included: ever use, current use, age at first and last use, total duration of use, constituents of each preparation used and duration of use of each preparation. Based on the information provided, HT preparations were classified as those containing oestrogen only, oestrogen-progestagen, or other/unknown types. A few women were recorded as having used both oestrogen-only and oestrogen-progestagen preparations and were classified by the preparation last use (ie, only 54 of the cases who were current or recent ex-users were recorded as having changed from use of oestrogen-only to oestrogen-progestagen HT; and only 150 were recorded as having changed from oestrogen-progestagen to oestrogen-only HT). Limited information was available about the specific constituents of the oestrogen-only and oestrogen-progestagen preparations used. For example, only about two-fifths of those who were recorded as having used combined oestrogen-progestagen preparations had information recorded on whether the progestagens had been used every day or less often. In most analyses current users were combined with recent ex-users who stopped <5 years previously, ie, "current-or-recent users". Follow-up in prospective studies was censored 4 years after HT use was last recorded and so the only possible source of misclassification in such analyses is if never-users start or if ex-users restart within 4 years. Data from one large prospective study suggest that such changes in use were relatively uncommon, in that before 2003 only 1% of never users became current users each year and 4% of past users became current users each year[38] (and after 2003 even fewer never and past users started HT). Sensitivity analyses explored cut-offs other than 4 years (Appendix p17).

Classification of ovarian cancers

All but 3 studies[15,16,23] contributed information about tumour subtype and most investigators provided pre-coded information on this. In the few studies that provided data coded to the International Classification of Diseases for Oncology (3rd edition. Eds: Fritz AG, Percy C, Jack A, et al. Geneva: World Health Organization; 2000), tumours were classified as epithelial (clear cell [M8310/8313], endometrioid [M8380], mucinous [M8470/8480/ 8490], serous [M8441/8460], and mixed, other or not otherwise specified [all other ICD10-O codes for epithelial tumours within ICD10 C56]); non-epithelial (M8620/8631/8650/8862/8890/8933/8951/8963/9080/ 9084/9110); or not specified as either epithelial or non-epithelial (M8000). Epithelial cancers with codes M8442/ 8451/8462/8472 were classified as being borderline-malignant.

Statistical Methods

When more than two groups were compared, the variance of the log risk was estimated for each group (Plummer M. Improved estimates of floating absolute risk. *Stat Med* 2004; 23: 93- 104) and these group-specific variances were used to calculate group-specific confidence intervals. This method yields valid comparisons between any two groups, even if neither is the baseline group. It allows the relative risk estimates to be treated as approximately independent in tests of heterogeneity and trend.

Estimates from stratified analyses were combined, weighted by the amount of statistical information in each stratum (inverse of the variance of log risk). Comparisons across different subgroups of women were made using standard chi-squared tests for heterogeneity, calculated from the change in log likelihood on adding extra terms.

References to 58 epidemiological studies of ovarian cancer that have collected information about HT use

1. Newhouse ML, Pearson RM, Fullerton JM, Boesen EAM, Shannon HS. A case-control study of carcinoma of the ovary. *British J Preventive Social Medicine* 1977; **31:** 148-53.

2. McGowan L, Parent L, Lednar W, Norris HJ. The woman at risk for developing ovarian cancer. *Gynecol Oncol* 1979; **7:** 325-44.

3. Hildreth NG, Kelsey JL, LiVolsi VA, Fischer DB, Holford TR, Mostow ED, Schwartz PE, White C. An epidemiologic study of epithelial carcinoma of the ovary. *Am J Epidemiol* 1981; **114:** 398-405.

4. Cramer DW, Welch WR, Scully RE, Wojciechowski CA. Ovarian cancer and talc: a case-control study. *Cancer* 1982; **50(2):** 372-76.

5. Weiss NS, Lyon JL, Krishnamurthy S, Dietert SE, Liff JM, Daling JR. Noncontraceptive estrogen use and the occurrence of ovarian cancer. *J Natl Cancer Inst* 1982; **68:** 95-98.

6. Byers T, Marshall J, Graham S Mettlin C, Swanson M. A case-control study of dietary and nondietary factors in ovarian cancer. *J Natl Cancer Inst* 1983; **71:** 681-86.

7. Nasca PC, Greenwald P, Chorost, et al. An epidemiologic case-control study of ovarian cancer and reproductive factors**.** *Am J Epidemiol* 1984; **119:** 705-13.

8. The Cancer And Steroid Hormone Study of the Centers for Disease Control and the National Institute of Child Health and Human Development. The reduction in risk of ovarian cancer associated with oral contraceptive use. *N Engl J Med* 1987; **316:** 650-55.

9. Hartge P, Hoover R, McGowan L, Lesher L, Norris HJ. Menopause and ovarian cancer. *Am J Epidemiol* 1988; **127:** 990-98.

10. Whittemore A, Wu M, Paffenbarger R, et al. Personal and environmental characteristics related to epithelial ovarian cancer. II Exposures to talcum powder, tobacco, alcohol and coffee. *Am J Epidemiol* 1988; **128:** 1228-40.

11. Wu ML, Whittemore AS, Paffenbarger RS, et al. Personal and environmental characteristics related to epithelial ovarian cancer. I Reproductive and menstrual events and oral contraceptive use. *Am J Epidemiol* 1988; **128:** 1216-27

12. Booth M, Beral V, Smith P. Risk factors for ovarian cancer: a case-control study. *Br J Cancer* 1989; **60:** 592-98.

13. Kaufmann DW, Kelly JP, Welch WR et al. Noncontraceptive estrogen use and epithelial ovarian cancer. *Am J Epidemiol* 1989; **130:** 1142-51.

14. The WHO Collaborative Study of Neoplasia and Steroid Contraceptives. Epithelial ovarian cancer and combined oral contraceptives. *Int J Epidemiol* 1989; **18:** 538-45.

15. Parazzini F, La Vecchia C, Negri E, Bocciolone L, Fedele L, Franceschi S. Oral contraceptive use and the risk of ovarian cancer: an Italian case-control study. *Eur J Cancer* 1991; **27:** 594-98.

16. Polychronopoulou A, Tzonou A, Hsieh CC, et al. Reproductive variables, tobacco, ethanol, coffee and somatometry as risk factors for ovarian cancer. *Int J Cancer* 1993; **55:** 402-07.

17. Risch HA. Estrogen replacement therapy and risk of epithelial ovarian cancer. *Gynecol Oncol J* 1996; **63:** 254-57

18. Hempling RE, Wong C, Piver MS, Natarajan N, Mettlin CJ. Hormone replacement therapy as a risk factor for epithelial ovarian cancer: results of a case-control study. *Obstet Gynecol.* 1997; **89:** 1012-16.

19. Mosgaard B, Lidegaard O, Kjaer S, Schou G, Andersen A. Infertility, fertility drugs, and invasive ovarian cancer: a case- control study. *Fertil Steril* 1997; **67:** 1005-12.

20. Purdie D, Bain C, Siskind V et al. Hormone replacement therapy and risk of epithelial ovarian cancer. *Br J Cancer* 1999; **81:** 559-63.

21. Cramer DW, Greenberg R, Titus-Ernstoff L, et al. A case-control study of galactose consumption and metabolism in relation to ovarian cancer. *Cancer Epidemiol Biomarkers & Prev.* 2000; **9:** 95-101.

22. Chiaffarino F, Pelucchi C, Parazzini F, et al. Reproductive and hormonal factors and ovarian cancer. *Ann Oncol* 2001; **12:** 337-41.

23. Rodriguez C, Patel AV, Calle EE, Jacob EJ, Thun MJ. Estrogen replacement therapy and ovarian cancer mortality in a large prospective study of US women. *JAMA* 2001; **285:** 1460-65.

24. Goodman MT, Wu AH, Tung K-H, et al. Association of dairy products, lactose and calcium with the risk of ovarian cancer. *Amer J Epidemiol* 2002; **156:** 148-57.

25. Lacey JV, Mink PJ, Lubin JH, et al. Menopausal hormone replacement therapy and risk of ovarian cancer. *JAMA* 2002; **288:** 334-41.

26. Riman T, Dickman PW, Nilsson S, et al. Hormone replacement therapy and risk of invasive epithelial ovarian cancer in Swedish women. *J Natl Cancer Inst.* 2002; **94:** 497-504.

27. Royer J, Becher H, Chang-Claude J. Low dose oral contraceptives: protective effect on ovarian cancer risk. *Int J Cancer* 2002; **95:** 370-74.

28. Sit AS, Modugno F, Weissfeld JL, Berga S, Ness NB. Hormone replacement therapy formulations and risk of epithelial ovarian carcinoma. *Gynecologic Oncology* 2002; **86,** 118-23.

29. McCann SE, Freudenheim JL, Marshall JR, Graham S. Risk of human ovarian cancer is related to dietary intake of selected nutrients, phytochemicals and food groups. *J Nutr* 2003; **133:** 1937-42.

30. Olsson HL, Ingvar C, Bladstrom A. Hormone replacement therapy containing progestins and given continuously increases breast carcinoma risk in Sweden. *Cancer* 2003; **97(6):** 1387-92.

31. Bakken K, Alsaker E, Eggen AE, Lund E. Hormone replacement therapy and incidence of hormone-dependent cancersin the Norwegian women and cancer study. *Int J Cancer* 2004; **112:** 130-34.

32. Folsom AR, Anderson JP, Ross JA. Estrogen replacement therapy and ovarian cancer. *Epidemiology* 2004; **15:** 100-4

33. Graff-Iversen S, Hammar N, Thelle DS, Tonstad S. Hormone therapy and mortality during 14-year follow-up of 14,324 Norwegian women. *J Inter Med* 2004; **256:** 1-9.

34. Pike MC, Pearce CL, Peters R, Cozen W, Wan P, Wu AH. Hormonal factors and the risk of invasive ovarian cancer: a population-based case-control study. *Fertility and Sterility* 2004; **82:** 186-95.

35. Rossing MA, Tang M-TC, Flagg EW, Weiss LK, Wicklund KG.A case-control study of ovarian cancer in relation to infertility and the use of ovulation inducing drugs. *Amer J Epidemiol* 2004; **160:** 1070-79.

36. Zhang M, Lee AH, Binns CW. Reproductive and dietary risk factors for epithelial ovarian cancer in China. *Gynecol Oncology* 2004; **93:** 320-26.

37. Peterson NB, Trentham-Dietz A, Newcomb PA, et al. Relation of anthropometric measurements to ovarian cancer risk in a population-based case-control study (United States). *Cancer Causes Control* 2006; **17:** 459-67.

38. Beral V, Reeves G, Green J, Bull D, for the Million Women Study Collaborators. Ovarian cancer and hormone replacement therapy in the Million Women Study. *Lancet* 2007; **369:** 1703-10.

39. Danforth KN, Tworogood SS, Hecht JL, et al. A prospective study of postmenopausal hormone use and ovarian cancer risk. *British J Cancer* 2007; **96:** 151-56.

40. Garcıa-Closas M, Brinton L, Lissowska J, et al. Ovarian cancer risk and common variation in the sex hormone-binding globulin gene: a population-based case–control study. *BMC Cancer* 2007; **7:** 60.

41. Silvera SAN, Jain M, Howe GR, Miller AR, Rohan TE. Dietary fiber intake and ovarian cancer risk: a prospective cohort study. *Cancer Causes Control* 2007; **18:** 335-41.

42. Gram IT, Braaten T, Adami HO, Lund E, Weiderpass E. Cigarette smoking and risk of borderline and invasive epithelial ovarian cancer. *Int J Cancer* 2008; **122:** 647-52.

43. Olsen CM, Nagle CM, Whiteman DC, et al. Australian Cancer Study (Ovarian Cancer), Australian Ovarian Cancer Study Group. Body size and risk of epithelial ovarian and related cancers: a population-based case-control study. *Int J Cancer* 2008; **123:** 450-56.

44. Larsson SC, Akesson A, Wolk A. Long-term dietary acrylamide intake and risk of epithelial ovarian cancer in a prospective cohort of Swedish women. *Cancer Epidemiol Biomarkers Prev* 2009; **18**: 994-97.

45. Mørch LS, Løkkegaard E, Andreasen AH, et al. Hormone therapy and ovarian cancer. *JAMA* 2009; **302:** 289-305.

46. Braem MGM, Onland-Moret NC, van den Brandt PA, et al. Reproductive and hormonal factors in association with ovarian cancer in the Netherlands Cohort Study. *Am J Epidemiol* 2010; **172:** 1181-89.

47. Hildebrand JS, Gapstur SM, Teras L, Patel AV. Postmenopausal hormone use and incident ovarian cancer: associations differ by regimen. *Int J Cancer* 2010; **127:** 2928-35.

48. Buys SS, Partridge E, Black A, et al. Effect of screening on ovarian cancer mortality: the Prostate, Lung, Colorectal and Ovarian (PLCO) Cancer Screening Randomized Controlled Trial. *JAMA* 2011; **305:** 2295-303.

49. Tsilidis KK, Allen NE, Key TJ, et al. Menopausal hormone therapy and risk of ovarian cancer in the European prospective investigation into cancer and nutrition. *Cancer Causes Control* 2011; **8:** 1075-84.

50. Lo-Ciganic WH, Zgibor JC, Bunker CH, Moysich KB, Edwards RP, Ness RB. Aspirin, nonaspirin nonsteroidal anti-inflammatory drugs, or acetaminophen and risk of ovarian cancer. *Epidemiology* 2012; **23(2):** 311-19.

51. Trabert B, Wentzensen N, Yng HP, et al. Ovarian cancer and menopausal hormone therapy in the NIH-AARP diet and health study. *Brit J Cancer* 2012; **107:** 1181-87.

52. Lee AH, Su D, Pasalich M, Binns CW. Tea consumption reduces ovarian cancer risk. *Cancer Epidemiol* 2013; **37**: 54-59.

53. Chetrit A, Hirsh-Yechezkel G, Ben-David Y, Lubin F, Friedman E, Sadetzki S. Effect of BRCA 1/2 mutations on long-term survival of patients with invasive ovarian cancer: the national Israeli study of ovarian cancer. *J Clin Oncol* 2008; **26(1):** 20-25.

54. Prentice RL et al, Conjugated Equine Estrogens and Breast Cancer Risk in the Women’s Health Initiative Clinical Trial and Observational Study *Am J Epid* 2008; **167:** 1407-15

55. Canchola AJ, Chang ET, Bernstein L, et al. Body size and the risk of ovarian cancer by hormone therapy use in the California Teachers Study cohort. *Cancer Causes Control* 2010; **21(12):** 2241-48.

56. Mills PK, Riordan DG, Cress RD, Goldsmith DF. Hormone replacement therapy and invasive and borderline epithelial ovarian cancer risk. *Cancer Detect Prev* 2005; **29:** 124-32.

57. Moorman PG, Schildkraut JM, Calingaert B, Halabi S, Berchuck A. Menopausal hormones and risk of ovarian cancer. *Am J Obstet Gynecol* 2005; **193:** 76-82.

58. Rossing MA, Cushing-Haugen KL, Wicklund KG, Doherty JA, Weiss NS. Menopausal hormone therapy and risk of epithelial ovarian cancer. *Cancer Epidemiol Biomarkers Prev* 2007 **16(12):** 2548-56.

Details of the 52 studies included in these analyses

	Postmenopausal cases/controls	Median year of Diagnosis (cases)	Mean age (cases)
**17 Studies with prospectively recorded data on HT use**			
BCDDP (USA) [25]	325/1267	1989	66.6
IOWA Women's Health[32]	91/330	1989	66.5
Norwegian Counties[33]	22/101	1990	58.5
Netherlands Cohort[46]	174/1151	1990	67.9
CPS-II Mortality (USA)[23]	1258/5542	1992	74.0
CNBSS (Canada)[41]	139/382	1993	62.8
Nurses' Health Study (USA) [39]	435/1836	1993	62.9
Southern Swedish[30]	28/116	1994	61.9
Swedish mammography[44]	131/575	1995	67.2
CPS-II Nutrition (USA) [47]	336/1346	1997	68.3
EPIC (8 countries)[49]	325/1373	1999	63.2
NIH-AARP(USA)[51]	381/1522	1999	66.6
DaHoRS (Denmark) [45]	2110/ -	2000	62.2
NOWAC (Norway)[31]	95/384	2000	59.7
PLCO (USA)[48]	197/786	2001	68.2
Million Women Study (UK)[38]	6022/23880	2005	64.2
WLH (Norway/Sweden)[42]	41/126	2005	57.9
**All prospective studies**	**12110/40,717***	**2000**	**65.1**
**35 studies with retrospectively recorded data on HT use**			
Byers (USA)[6†]	39/286	1958	59.1
Newhouse (UK)[1†]	151/321	1973	62.2
McGowan (USA)[2†]	60/59	1975	58.9
Paffenbarger (USA)[11†]	53/253	1975	66.1
Weiss (USA)[5]	187/565	1977	61.6
Hildreth/Kelsey (USA)[3†]	51/769	1978	62.4
Nasca (USA)[7]	224/394	1978	62.5
Cramer I (USA)[4]	127/116	1979	62.5
Booth (UK)[12†]	146/243	1980	56.9
Hartge (USA)[9†]	152/181	1980	62.2
CASH (USA)[8]	89/758	1981	50.8
WHO developed countries (Australia, Israel)[14†]	34/235	1982	49.8
Rosenberg (USA)[13†]	467/1807	1983	60.1
Whittemore (USA)[10]	102/266	1984	60.8
Negri/Franceschi (Italy)[15†]	550/1395	1986	61.1
Western New York (USA)[29]	81/404	1988	64.1
PEDS (USA)[18†]	230/979	1990	63.7
Tzonou/Tricopoulos (Greece) [16†]	244/284	1990	61.2
Risch (Canada)[17]	305/364	1991	62.4
Green/Purdie (Australia)[20]	487/488	1992	63.7
Mosgaard (Denmark)[19]	322/343	1992	54.1
Cramer II (USA)[21]	258/222	1993	62.3
Riman (Sweden)[26]	677/3453	1994	63.5
German OCS[27]	179/321	1995	62.3
Pike/Wu (USA)[34]	304/344	1995	62.1
Negri/La Vecchia (Italy)[22†]	657/1526	1995	61.6
Goodman/Wu (USA)[24]	403/498	1996	64.9
SHARE (USA)[28]	399/599	1996	59.7
OVCARE (USA)[35]	50/149	1997	50.3
Newcomb (Two States;USA)[37]	298/1685	1998	62.8
Zhejiang-Curtin (China)[36†]	89/226	1999	59.4
Polish study (Poland)[40]	179/1283	2002	62.6
AOCS (Australia)[43]	913/945	2004	63.5
HOPE (USA)[50]	410/924	2005	64.6
Guangzhou (China)[52†]	461/444	2006	59.5
**All retrospective studies**	**9378/23,129**	**1994**	**61.7**
**All 52 studies**	**21,488/63,846***	**1999**	**63.6**

* Excludes DaHoRS[45]

† Retrospective case-control studies with hospital controls

Estimated absolute risk of ovarian cancer in HT users

As an example, we estimated the absolute excess risk of ovarian cancer associated with 5 years and 10 years use of HT, starting at age 50 for women in England. We used age-specific ovarian cancer rates in England for 2000-2003 (National Cancer Statistics for England http://www.ons.gov.uk/ons/taxonomy/index.html?nscl=Cancer; accessed 23 June 2013), data on the prevalence of HT use in 2000-2003 (Watson J et al. *Eur J Clin Pharmacology* 2007; 63: 843-49) and the relative risks found here in prospective studies (Figure 1) to estimate incidence rates in never users of HT (table below). National rates for ovarian cancer include a relatively small proportion of women who had had an oophorectomy in the denominator and, for comparability, the population at risk for these calculations also include women who had had an oophorectomy.

The relative risks for HT-associated incident and fatal ovarian cancer are similar.[23,38] To estimate risk of fatal disease it was assumed that 65% of the ovarian cancers were fatal, since 5-year survival in European countries was estimated to be 30-40% during the 1990s and 2000s (Karim-Kos HE et al. Recent trends of cancer in Europe. *Eur J Cancer* 2008; 44: 1345-89) and 10-year survival in the US SEER data was estimated to be 35% (Kosary CL. Cancer of the ovary: SEER survival monograph. Bethesda: National Cancer Institute; 2007. http://seer.cancer.gov/publications/survival/surv_ovary.pdf, accessed 23 June 2014).

HT use for 5 years, starting at age 50 in England, is estimated to result in one additional ovarian cancer in every 1000 HT users, and one additional death from the disease in every 1700 users; and use for 10 years is estimated to result in one additional ovarian cancer in every 600 HT users, and one additional death from the disease in every 800 users (table below).

Ovarian cancer incidence in other high income countries is similar to that in England (International Agency for Research on Cancer. http://www-dep.iarc.fr, accessed June 23, 2014), so the excess risks calculated here would be broadly similar for women in other high income countries.

Estimated excess incidence of ovarian cancer in England associated with 5 years and 10 years use of HT, starting at age 50 years

	**5-year risk of ovarian cancer per thousand women who never used HT** *	**Excess risk of ovarian cancer associated with 5 years of HT use, starting from age 50 years**		**Excess risk of ovarian cancer associated with 10 years of HT use, starting from age 50 years**	
		**Proportional excess risk (RR-1)** †	**Absolute 5-year excess per 1000 HT users**	**Proportional excess risk (RR-1)** †	**Absolute 5-year excess per 1000 HT uses**
Age 50-54	1.2	0.43	0.52	0.43	0.52
Age 55-59	1.6	0.23	0.37	0.42	0.67
Age 60-64	2.1	0.05	0.10	0.29	0.61
**Excess incidence**			**0.99 per 1000; 1 in 1000 users**		**1.80 per 1000; 1 in 600 users**
**Excess deaths**			**0.6 per 1000; 1 in 1700 users**		**1.2 per 1000; 1 in 800 users**

† From relative risk (RR) estimates in Figure 2 (for women who use HT for 5 years, the average duration of use in current users is 2.5 years; and the average duration of use in ex-users is 5 years; for women who use HT for 10 years, the average duration of use in current users is 2.5 years in the first 5 years and 7.5 years in the next 5 years, and the average duration of use in ex-users is 10 years)

WEBFIGURES

Webfigure 1. Study-specific results for the relative risk of ovarian cancer for ever users versus never-users of HT.

Study (Country)[ref]	Number of cases in ever users/ never-users of HT	Relative risk for ever users vs never-users of HT (99% CI)*
**PROSPECTIVE STUDIES:**		
**Europe**		
Norwegian Counties[33]	2/20	1.15 (0.13-10.17)
Netherlands Cohort[46]	26/148	1.02 (0.55-1.88)
Southern Swedish[30]	8/20	1.09 (0.27-4.42)
Swedish Mammography[44]	71/60	0.89 (0.50-1.57)
EPIC (8 countries)[49]	185/140	1.33 (0.91-1.95)
DaHoRS (Denmark)[45]	726/1384	1.34 (1.18-1.52)
NOWAC (Norway)[31]	35/60	1.11 (0.55-2.24)
Million Women Study (UK)[38]	3522/2500	1.15 (1.06-1.26)
WLH (Norway/Sweden)[42]	26/15	1.00 (0.29-3.46)
**All Europe**	**4601/4347**	**1.21 (1.12-1.29)**
**North America**		
BCDDP (USA)[25]	205/120	1.25 (0.87-1.79)
IOWA Women's Health (USA)[32]	59/32	1.17 (0.56-2.48)
CPS-II - Mortality (USA)[23]	773/485	1.03 (0.85-1.25)
Nutrition (USA)[47]	220/116	1.44 (1.02-2.03)
CNBSS (Canada)[41]	116/23	1.58 (0.61-4.08)
Nurses' Health Study (USA)[39]	258/177	1.22 (0.90-1.64)
NIH - AARP (USA)[51]	224/157	1.50 (1.00-2.24)
PLCO (USA)[48]	145/52	1.42 (0.79-2.56)
**All North America**	**2000/1162**	**1.20 (1.06-1.36)**
**ALL PROSPECTIVE STUDIES**	**6601/5509**	**1.20 (1.13-1.28)**
**RETROSPECTIVE STUDIES:**		
**Europe**		
Newhouse (UK)[1]	19/132	0.52 (0.23-1.19)
Booth (UK)[12]	24/122	1.24 (0.56-2.76)
Negri/Franceschi (Italy)[15]	47/503	1.77 (1.04-3.02)
Tzonou/Trichopoulos (Greece)[16]	14/230	2.01 (0.68-5.96)
Mosgaard (Denmark)[19]	142/180	0.86 (0.56-1.31)
Riman (Sweden)[26]	209/468	1.26 (0.97-1.62)
German OCS[27]	45/134	0.81 (0.44-1.51)
Negri/La Vecchia (Italy)[22]	53/604	1.44 (0.87-2.41)
Polish Study (Poland)[40]	41/138	1.19 (0.67-2.11)
**All Europe**	**594/2511**	**1.17 (1.00-1.38)**
**North America**		
Byers (USA)[6]	5/34	0.63 (0.15-2.64)
McGowan (USA)[2]	20/40	1.38 (0.32-5.96)
Paffenbarger (USA)[11]	37/16	1.04 (0.41-2.67)
Weiss (USA)[5]	104/83	1.21 (0.73-2.00)
Hildreth/Kelsey (USA)[3]	14/37	1.00 (0.41-2.45)
Nasca (USA)[7]	68/156	0.97 (0.58-1.62)
Cramer I (USA)[4]	22/105	0.99 (0.38-2.59)
Hartge (USA)[9]	49/103	0.72 (0.38-1.38)
CASH (USA)[8]	36/53	0.86 (0.46-1.63)
Rosenberg (USA)[13]	90/377	1.26 (0.86-1.85)
Whittemore (USA)[10]	62/40	1.14 (0.56-2.29)
Western New York (USA)[29]	18/63	0.88 (0.39-2.02)
PEDS (USA)[18]	39/191	0.74 (0.44-1.25)
Risch (Canada)[17]	76/229	1.28 (0.75-2.20)
Cramer II (USA)[21]	54/204	1.45 (0.67-3.12)
Pike/Wu (USA)[34]	173/131	0.99 (0.63-1.56)
Goodman/Wu (USA)[24]	218/185	0.67 (0.44-1.02)
SHARE (USA)[28]	129/270	0.97 (0.65-1.47)
OVCARE (USA)[35]	26/24	1.37 (0.38-4.90)
Newcomb (Two States;USA)[37]	124/174	1.09 (0.77-1.57)
HOPE (USA)[50]	203/207	0.87 (0.61-1.26)
**All North America**	**1567/2722**	**0.99 (0.87-1.11)**
**Elsewhere**		
WHO developed countries (Australia/Israel)[14]	5/29	0.97 (0.21-4.38)
Green/Purdie (Australia)[20]	102/385	0.89 (0.58-1.37)
Zheijang-Curtin (China)[36]	1/88	0.42 (0.02-7.42)
AOCS (Australia)[43]	426/487	0.86 (0.66-1.11)
Guangzhou (China)[52]	7/454	2.46 (0.40-15.20)
**All elsewhere**	**541/1443**	**0.88 (0.70-1.09)**
**ALL RETROSPECTIVE STUDIES**	**2702/6676**	**1.02 (0.93-1.11)**
**ALL STUDIES**	**9303/12185**	**1.14 (1.09-1.20)**

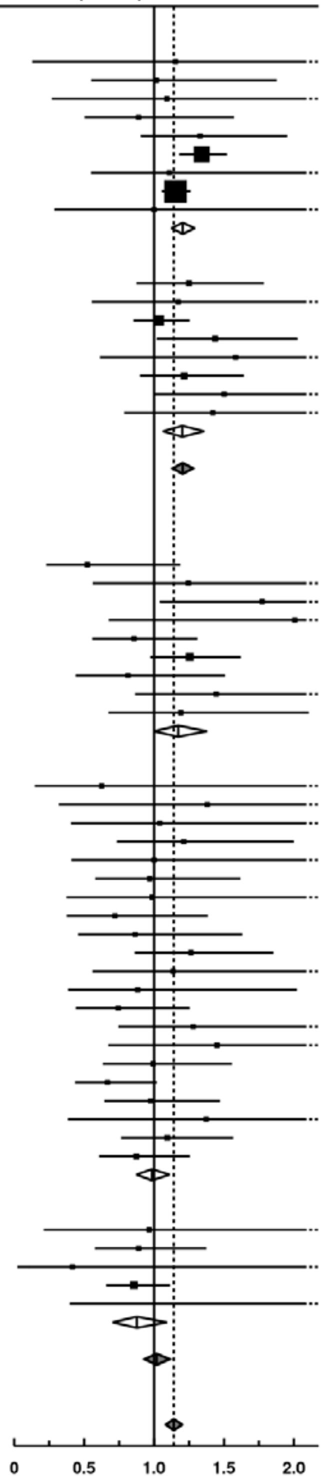

* Risk relative to never-users of HT, stratified by age at diagnosis, study and body mass index, and adjusted for age at menopause, hysterectomy, oral contraceptive use and parity.

The dotted line represents the overall result for all studies.

Webfigure 2. Study-specific results for the relative risk of ovarian cancer for ever users versus never-users of HT in women with information on duration of use and time since last use of HT.

Study (Country)[ref]	Number of cases in ever users/ never-users of HT	Relative risk for ever users vs never-users of HT (99% CI)*
**PROSPECTIVE STUDIES:**		
**Europe**		
Norwegian Counties[33]		no data
Netherlands Cohort[46]	19/148	0.94 (0.47-1.88)
Southern Swedish[30]	3/20	1.05 (0.16-6.98)
Swedish Mammography[44]		no data
EPIC (8 countries)[49]	63/140	1.58 (0.91-2.74)
DaHoRS (Denmark)[45]	625/1384	1.43 (1.26-1.62)
NOWAC (Norway)[31]	33/60	1.16 (0.55-2.42)
Million Women Study (UK)[38]	2453/2500	1.16 (1.05-1.28)
WLH (Norway/Sweden)[42]	15/15	0.96 (0.23-3.97)
**All Europe**	**3211/4267**	**1.26 (1.17-1.35)**
**North America**		
BCDDP (USA)[25]	159/120	1.28 (0.87-1.88)
IOWA Women's Health (USA)[32]	1/32	insufficient data
CPS-II - Mortality (USA)[23]	494/485	1.03 (0.83-1.28)
Nutrition (USA)[47]	91/116	1.57 (0.99-2.51)
CNBSS (Canada)[41]	41/23	1.25 (0.40-3.88)
Nurses' Health Study (USA)[39]	210/177	1.15 (0.84-1.57)
NIH - AARP (USA)[51]	153/157	1.48 (0.94-2.34)
PLCO (USA)[48]	59/52	1.04 (0.44-2.43)
**All North America**	**1208/1162**	**1.17 (1.01-1.35)**
**ALL PROSPECTIVE STUDIES**	**4419/5429**	**1.24 (1.16-1.32)**
**RETROSPECTIVE STUDIES:**		
**Europe**		
Newhouse (UK)[1]		no data
Booth (UK)[12]		no data
Negri/Franceschi (Italy)[15]	45/503	1.76 (1.02-3.02)
Tzonou/Trichopoulos (Greece)[16]		no data
Mosgaard (Denmark)[19]		no data
Riman (Sweden)[26]	203/468	1.26 (0.98-1.64)
German OCS[27]	45/134	0.81 (0.44-1.51)
Negri/La Vecchia (Italy)[22]	52/604	1.43 (0.86-2.40)
Polish Study (Poland)[40]	29/138	0.99 (0.51-1.94)
**All Europe**	**374/1847**	**1.26 (1.04-1.53)**
**North America**		
Byers (USA)[6]		no data
McGowan (USA)[2]		no data
Paffenbarger (USA)[11]	16/16	0.55 (0.15-2.04)
Weiss (USA)[5]	101/83	1.20 (0.72-1.98)
Hildreth/Kelsey (USA)[3]	13/37	1.30 (0.51-3.32)
Nasca (USA)[7]	38/156	0.99 (0.51-1.95)
Cramer I (USA)[4]	20/105	0.86 (0.32-2.33)
Hartge (USA)[9]	35/103	0.79 (0.37-1.68)
CASH (USA)[8]	11/53	1.06 (0.40-2.82)
Rosenberg (USA)[13]	77/377	1.18 (0.79-1.77)
Whittemore (USA)[10]	37/40	0.89 (0.39-2.03)
Western New York (USA)[29]		no data
PEDS (USA)[18]		no data
Risch (Canada)[17]	74/229	1.27 (0.74-2.19)
Cramer II (USA)[21]	54/204	1.45 (0.67-3.12)
Pike/Wu (USA)[34]	173/131	0.99 (0.63-1.56)
Goodman/Wu (USA)[24]	218/185	0.67 (0.44-1.02)
SHARE (USA)[28]	129/270	0.98 (0.65-1.47)
OVCARE (USA)[35]	26/24	1.37 (0.38-4.91)
Newcomb (Two States;USA)[37]	124/174	1.11 (0.77-1.58)
HOPE (USA)[50]	203/207	0.88 (0.61-1.26)
**All North America**	**1349/2394**	**1.00 (0.88-1.14)**
**Elsewhere**		
WHO developed countries (Australia/Israel)[14]	4/29	1.01 (0.21-4.95)
Green/Purdie (Australia)[20]	81/385	0.92 (0.57-1.49)
Zheijang-Curtin (China)[36]		no data
AOCS (Australia)[43]	404/487	0.87 (0.67-1.14)
Guangzhou (China)[52]		no data
**All elsewhere**	**489/901**	**0.89 (0.70-1.12)**
**ALL RETROSPECTIVE STUDIES**	**2212/5142**	**1.04 (0.94-1.15)**
**ALL STUDIES**	**6631/10571**	**1.17 (1.11-1.24)**

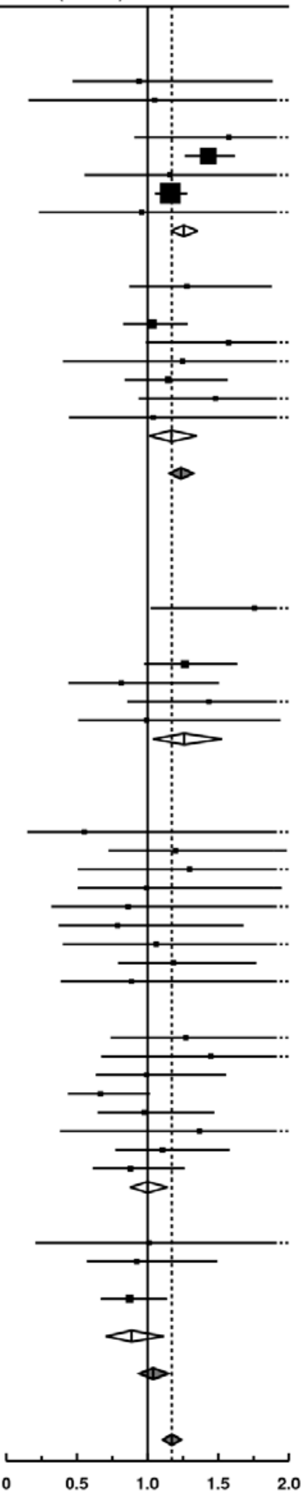

* Risk relative to never-users of HT, stratified by age at diagnosis, study and body mass index, and adjusted for age at menopause, hysterectomy, oral contraceptive use and parity.

The dotted line represents the overall result for all studies.

ovarianHR1

Webfigure 3. Study-specific results for the relative risk of ovarian cancer for current-or-recent users versus never-users of HT.

Study (Country)[ref]	Number of cases in current-or-recent users/ never-users of HT	Relative risk for current-or-recent users vs never-users of HT (99% CI)*
**PROSPECTIVE STUDIES:**		
**Europe**		
Norwegian Counties[33]		no data
Netherlands Cohort[46]	0/148	insufficient data
Southern Swedish[30]	1/20	insufficient data
Swedish Mammography[44]		no data
EPIC (8 European countries)[49]	57/140	1.71 (0.96-3.06)
DaHoRS (Denmark)[45]	600/1384	1.49 (1.31-1.69)
NOWAC (Norway)[31]	25/60	1.13 (0.49-2.58)
Million Women Study (UK)[38]	1500/2500	1.28 (1.14-1.44)
WLH (Norway/Sweden)[42]	13/15	1.13 (0.25-5.08)
**All Europe**	**2196/4267**	**1.37 (1.26-1.50)**
**North America**		
BCDDP (USA)[25]	85/120	1.40 (0.87-2.24)
IOWA Women's Health (USA)[32]	1/32	insufficient data
CPS-II - Mortality (USA)[23]	23/485	1.21 (0.61-2.40)
Nutrition (USA)[47]	91/116	1.57 (0.99-2.51)
CNBSS (Canada)[41]	5/23	5.37 (0.48-60.61)
Nurses' Health Study (USA)[39]	173/177	1.17 (0.84-1.63)
NIH-AARP (USA)[51]	135/157	1.69 (1.04-2.74)
PLCO (USA)[48]	42/52	1.05 (0.37-2.94)
**All North America**	**555/1162**	**1.35 (1.11-1.65)**
**ALL PROSPECTIVE STUDIES**	**2751/5429**	**1.37 (1.27-1.48)**
**RETROSPECTIVE STUDIES:**		
**Europe**		
Newhouse (UK)[1]		no data
Booth (UK)[12]		no data
Negri/Franceschi (Italy)[15]	11/503	2.05 (0.71-5.93)
Tzonou/Trichopoulos (Greece)[16]		no data
Mosgaard (Denmark)[19]		no data
Riman (Sweden)[26]	145/468	1.29 (0.96-1.74)
German OCS[27]	39/134	0.79 (0.40-1.54)
Negri/La Vecchia (Italy)[22]	21/604	1.93 (0.86-4.33)
Polish Study (Poland)[40]	18/138	0.97 (0.44-2.15)
**All Europe**	**234/1847**	**1.25 (0.99-1.59)**
**North America**		
Byers (USA)[6]		no data
McGowan (USA)[2]		no data
Paffenbarger (USA)[11]	12/16	0.59 (0.14-2.41)
Weiss (USA)[5]	67/83	1.21 (0.68-2.13)
Hildreth/Kelsey (USA)[3]	8/37	1.16 (0.37-3.61)
Nasca (USA)[7]	19/156	1.12 (0.46-2.72)
Cramer I (USA)[4]	7/105	1.16 (0.22-5.96)
Hartge (USA)[9]	16/103	0.60 (0.23-1.60)
CASH (USA)[8]	5/53	0.60 (0.16-2.24)
Rosenberg (USA)[13]	51/377	1.21 (0.75-1.96)
Whittemore (USA)[10]	26/40	1.13 (0.45-2.83)
Western New York (USA)[29]		no data
PEDS (USA)[18]		no data
Risch (Canada)[17]	37/229	1.33 (0.64-2.78)
Cramer II (USA)[21]	38/204	1.77 (0.72-4.39)
Pike/Wu (USA)[34]	149/131	1.02 (0.64-1.63)
Goodman/Wu (USA)[24]	173/185	0.60 (0.38-0.94)
SHARE (USA)[28]	97/270	0.98 (0.63-1.52)
OVCARE (USA)[35]	25/24	1.28 (0.35-4.75)
Newcomb (Two States;USA)[37]	113/174	1.18 (0.81-1.72)
HOPE (USA)[50]	121/207	0.91 (0.60-1.40)
**All North America**	**964/2394**	**1.01 (0.87-1.17)**
**Elsewhere**		
WHO developed countries (Australia/Israel)[14]	2/29	insufficient data
Green/Purdie (Australia)[20]	49/385	0.84 (0.46-1.52)
Zheijang-Curtin (China)[36]		no data
AOCS (Australia)[43]	289/487	0.93 (0.69-1.25)
Guangzhou (China)[52]		no data
**All elsewhere**	**340/901**	**0.90 (0.69-1.18)**
**ALL RETROSPECTIVE STUDIES**	**1538/5142**	**1.04 (0.93-1.16)**

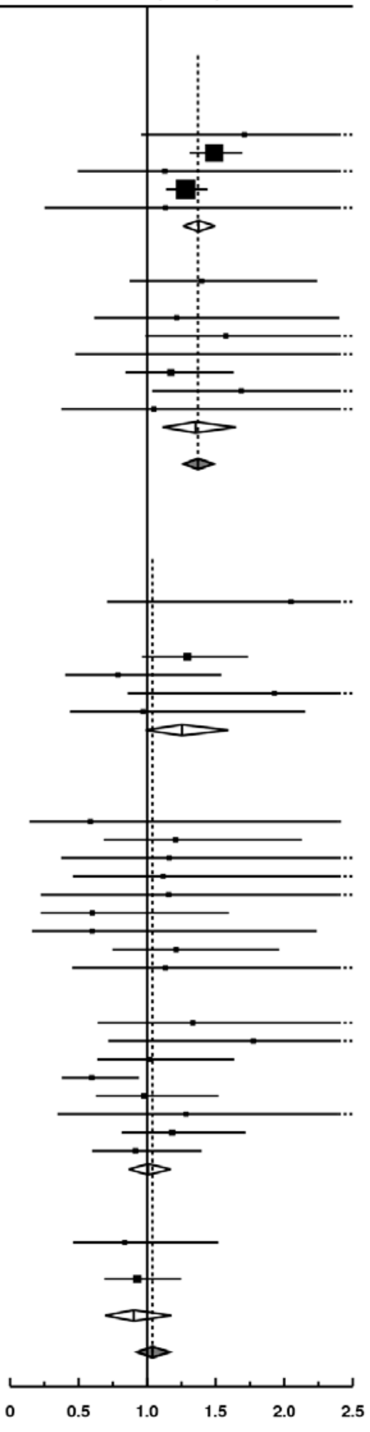

* Risk relative to never-users of HT, stratified by age at diagnosis, study and body mass index, and adjusted for age at menopause, hysterectomy, oral contraceptive use and parity.

Dotted lines represent totals for all prospective studies and, separately, for all retrospective studies.

ovarianHRTWfig3: 19/06

Webfigure 4: Relative risk of ovarian cancer for current-or-recent users compared to never-users of HT, by subgroup in prospective studies.

	Cases/Controls (current-or-recent users)	Relative risk for current-or-recent users compared to never-users of HT (95% CI)*
**All women**	2151/7436	1.31 (1.21-1.41)
**Age at menarche**		
<13	874/2864	1.31 (1.16-1.48)
≥13	1241/4453	1.31 (1.19-1.44)
χ^2 for heterogeneity=0.0002, p=0.99		
**Parity**		
0-2	1360/4321	1.26 (1.15-1.39)
≥3	671/2759	1.34 (1.18-1.53)
χ^2 for heterogeneity=0.56, p=0.5		
**Oral contraceptive use**		
never	993/2895	1.37 (1.23-1.52)
ever	1048/4097	1.26 (1.12-1.42)
χ^2 for heterogeneity=0.97, p=0.3		
**Height**		
<165 cm	1179/4428	1.30 (1.18-1.44)
≥165 cm	945/2907	1.32 (1.17-1.49)
χ^2 for heterogeneity=0.02, p=0.9		
**Body mass index**		
<25 kg/m^2	1091/3652	1.39 (1.25-1.55)
≥25 kg/m^2	984/3506	1.26 (1.14-1.41)
χ^2 for heterogeneity=1.43, p=0.2		
**Alcohol use**		
none	480/1667	1.44 (1.23-1.68)
any	1551/5403	1.28 (1.17-1.41)
χ^2 for heterogeneity=1.60, p=0.2		
**Tobacco use**		
never	1062/3523	1.40 (1.25-1.56)
ever	965/3561	1.25 (1.11-1.40)
χ^2 for heterogeneity=1.94, p=0.2		
**Mother or sister with ovarian/breast cancer**		
no	1752/6344	1.30 (1.19-1.41)
yes	259/644	1.50 (1.19-1.91)
χ^2 for heterogeneity=1.34, p=0.2		
**Hysterectomy†**		
no	187/819	1.26 (1.04-1.52)
yes	550/1451	1.51 (1.28-1.78)
χ^2 for heterogeneity=1.97, p=0.2		

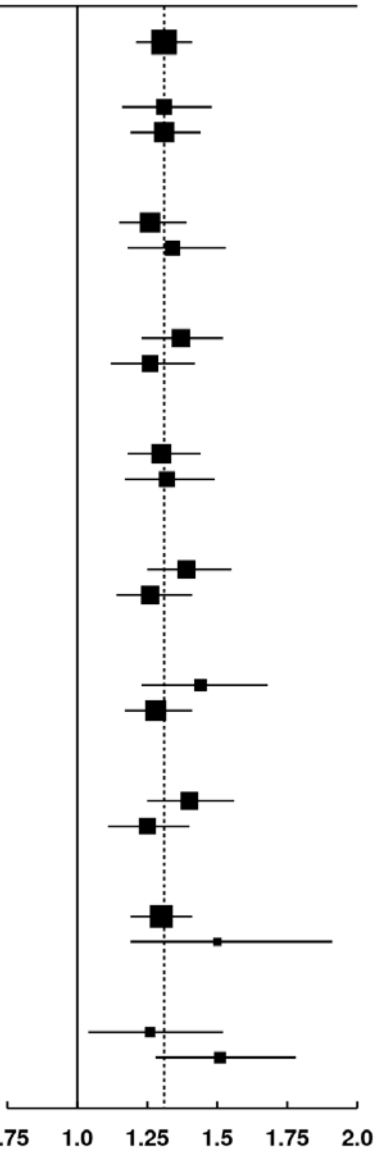

* Risk relative to never-users of HT, stratified by age at diagnosis, study and body mass index, and adjusted for age at menopause, hysterectomy, oral contraceptive use and parity. Results from DaHoRS were not available.

The dotted line represents the overall result for all women.

† Users of oestrogen-only HT, as few users of oestrogen-progestagen HT also had a hysterectomy.

ovarianHRTWfig4: 19/06/14

Webfigure 5: Relative risk of ovarian cancer for current-or-recent users compared to never-users of HT, by subgroup.

	Cases/Controls (current-or-recent users)	Relative risk for current-or-recent users compared to never-users of HT (95% CI)*
**All women**	3568/10626	1.18 (1.12-1.26)
**Age at menarche**		
<13	1408/4002	1.19 (1.08-1.32)
≥13	2033/6243	1.21 (1.12-1.31)
χ^2 for heterogeneity=0.04, p=0.8		
**Parity**		
0-2	2216/6079	1.16 (1.07-1.25)
≥3	1226/4121	1.22 (1.11-1.35)
χ^2 for heterogeneity=0.70, p=0.4		
**Oral contraceptive use**		
never	1672/4305	1.25 (1.16-1.36)
ever	1697/5614	1.14 (1.04-1.26)
χ^2 for heterogeneity=2.22, p=0.14		
**Height**		
<165 cm	1960/6248	1.17 (1.08-1.26)
≥165 cm	1526/4178	1.26 (1.15-1.38)
χ^2 for heterogeneity=1.44, p=0.2		
**Body mass index**		
<25 kg/m^2	1751/5097	1.31 (1.21-1.43)
≥25 kg/m^2	1568/5047	1.10 (1.01-1.19)
χ^2 for heterogeneity=8.75, p=0.003		
**Alcohol use**		
none	1024/2666	1.19 (1.06-1.33)
any	2271/7110	1.21 (1.13-1.31)
χ^2 for heterogeneity=0.10, p=0.8		
**Tobacco use**		
never	1795/5057	1.24 (1.14-1.34)
ever	1629/5136	1.14 (1.05-1.25)
χ^2 for heterogeneity=1.54, p=0.2		
**Mother or sister with ovarian/breast cancer**		
no	2873/9087	1.17 (1.10-1.25)
yes	496/1003	1.33 (1.11-1.59)
χ^2 for heterogeneity=1.55, p=0.2		
**Hysterectomy**†		
no	491/1515	1.22 (1.07-1.38)
yes	907/1970	1.44 (1.25-1.65)
χ^2 for heterogeneity=3.14, p=0.08		

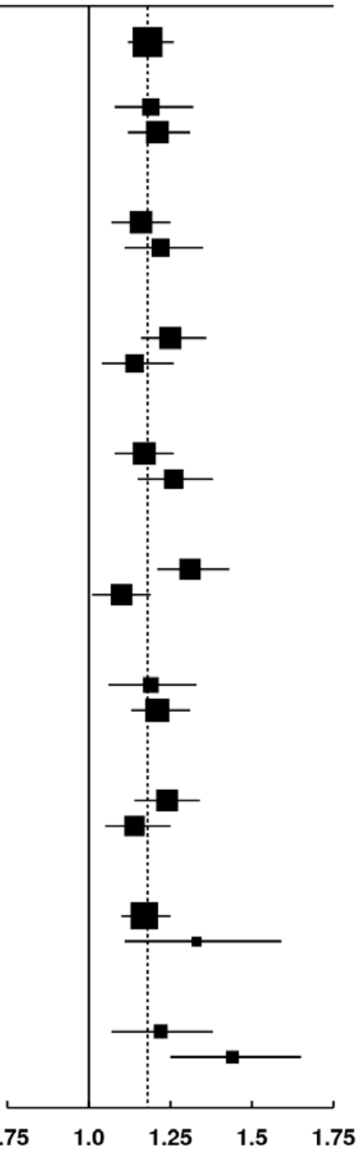

* Risk relative to never-users of HT, stratified by age at diagnosis, study and body mass index, and adjusted for age at menopause, hysterectomy, oral contraceptive use and parity. Retrospective studies with hospital controls are excluded and subgroup results from DaHoRS were not available.

The dotted line represents the overall result for all women.

† Users of oestrogen-only HT, as few users of oestrogen-progestagen HT also had a hysterectomy.

ovarianHRTWfig5: 19/06/14

WEBTABLES

Relative risk of ovarian cancer by duration and time since last HT use in retrospective studies only (see Figure 2 for results in prospective studies only, and in all studies combined)

	Median duration of HT use	Exposed cases (n)	Relative risk (95%CI)†
**Never users**	-	5142	1.00 (0.95-1.05)
**Current users**			
Duration <5 years	2	326	0.91 (0.79-1.05)
Duration ≥5 years	10	513	1.07 (0.96-1.20)
**Past users, ceased <5 years previously**			
Duration <5 years	1	300	1.07 (0.91-1.24)
Duration ≥5 years	10	399	1.26 (1.09-1.45)
**Past users, ceased ≥5 years previously**			
Duration <5 years	1	513	1.04 (0.93-1.17)
Duration ≥5 years	8	161	1.10 (0.90-1.35)

*Relative risk, stratified by study, centre within study, age and body mass index, and adjusted for parity, use of oral contraceptives, age at menopause and hysterectomy.

Relative risk of ovarian cancer in current-or-recent versus never users by study design and HT preparation last used

	Exposed cases (n)	Relative risk (95%CI)†
**Prospective studies**		
Oestrogen-only	1130	1.37 (1.26-1.50)
Oestrogen and progestagen	1167	1.37 (1.26-1.48)
**Retrospective studies**		
Oestrogen-only	742	1.21 (1.07-1.37)
Oestrogen and progestagen	498	0.96 (0.83-1.10)
**All studies**		
Oestrogen-only	1872	1.32 (1.23-1.41)
Oestrogen and progestagen	1665	1.25 (1.16-1.34)

†Relative risks were stratified by study, centre within study, age and body mass index, and adjusted for parity, use of oral contraceptives, age at menopause and hysterectomy.

Sensitivity analyses in prospective studies

Effect on the main findings of additional adjustment by various factors (not including DaHoRS[45])

	Relative risk (95%CI) in current-or-recent users vs never users of HT
Stratified by study, centre within study, age, and body mass index and adjusted for parity, past use of oral contraceptives, age at menopause, and hysterectomy (as in the main analyses)	**1.31 (1.21-1.41)**
**As above, and with additional adjustment for:**	
Year of birth	1.31 (1.22-1.41)
Ethnic origin	1.30 (1.21-1.40)
Years of education	1.31 (1.21-1.41)
Age at menarche	1.31 (1.21-1.41)
Height	1.31 (1.21-1.41)
Alcohol consumption	1.31 (1.21-1.41)
Smoking	1.32 (1.22-1.42)
First degree relative with ovarian or breast cancer	1.33 (1.23-1.43)
**Additional adjustment by all the above**	**1.33 (1.23-1.44)**

Effect of truncating follow-up at different times on the risk of ovarian cancer in current users

	HT duration of use in current vs never users of HT	
	<5 years<br>Relative risk† (95%CI)	≥5 years<br>Relative risk† (95%CI)
Truncating at 4 yrs *	1.43 (1.31-1.56)	1.39 (1.32-1.46)
Truncating at 3 yrs	1.42 (1.31-1.55)	1.39 (1.31-1.47)
Truncating at 2 yrs	1.44 (1.32-1.58)	1.43 (1.34-1.52)
Truncating at 1 yr	1.44 (1.31-1.58)	1.44 (1.34-1.54)

*as in the main analysis

Effect of truncating follow-up at different times on the relative risk in current-or-recent users

	Time from last report of HT use to cancer diagnosis	Number of exposed cases	Relative risk in current-or-recent vs never users of HT† (95%CI)
Truncating at 4 yrs*	1.6 years	2751	1.37 (1.29-1.46)
Truncating at 3 yrs	1.2 years	2487	1.37 (1.29-1.45)
Truncating at 2 yrs	0.9 years	2202	1.38 (1.30-1.47)
Truncating at 1 yr	0.6 years	1773	1.37 (1.28-1.47)
No truncation	3.3 years	3670	1.34 (1.27-1.41)

* as in the main analyses

Effect of excluding certain studies from the results

	Relative risk in current-or-recent users† (95%CI)
**All prospective studies**	1.37 (1.29-1.46)
Excluding both Million Women Study [38] and DaHoRS [45]	1.36 (1.19-1.56)
Excluding CPS mortality study[23]	1.37 (1.29-1.46)

†Relative risks were stratified by study, centre within study, age and body mass index, and adjusted for parity, use of oral contraceptives, age at menopause and hysterectomy.

Relative risk of ovarian cancer in current-or-recent versus never users of HT, by tumour characteristics:

By tumour histology (if known) and study design

	Exposed cases (n)	Relative risk (95%CI)†
**Prospective studies**		
Serous tumours	1286	1.53 (1.40-1.66); p<0.0001
Endometrioid tumours	298	1.42 (1.20-1.67); p<0.0001
Mucinous tumours	203	0.93 (0.77-1.12); p=0.4
Clear cell tumours	92	0.75 (0.57-0.98); p=0.04
Heterogeneity: p<0.0001		
**Retrospective studies**		
Serous tumours	922	1.20 (1.07-1.33); p= 0.001
Endometrioid tumours	210	1.10 (0.91-1.33); p=0.3
Mucinous tumours	100	0.59 (0.46-0.76); p<0.0001
Clear cell tumours	80	0.81 (0.60-1.10); p=0.2
Heterogeneity: p<0.0001		

By tumour histology and malignant potential of the tumour (if both known)

	Exposed cases (n)	Relative risk (95%CI)†
**Serous tumours**		
Fully malignant	1693	1.41 (1.32-1.51)
Borderline malignant	169	1.26 (1.01-1.58)
**Endometrioid tumours**		
Fully malignant	398	1.28 (1.13-1.45)
Borderline malignant	6	1.20 (0.33-4.37)
**Mucinous tumours**		
Fully malignant	154	0.81 (0.67-0.97)
Borderline malignant	102	0.73 (0.57-0.95)
**Clear cell tumours**		
Fully malignant	149	0.80 (0.65-0.98)
Borderline malignant	0	-

†Relative risk in current-or-recent versus never users of HT, stratified by study, centre within study, age and body mass index, and adjusted for parity, use of oral contraceptives, age at menopause and hysterectomy.

Relative risk of ovarian cancer in current or recent ex-users vs never-users of HT, by study design age at first use of HT (not including DaHoRS[45])

	Exposed cases (n)	Relative risk (95%CI)†
**Prospective studies**		
First use before age 50	826	1.35 (1.24-1.47)
First use at age 50-59	1070	1.31 (1.22-1.40)
First use at age ≥60	110	1.15 (0.93-1.43)
Heterogeneity: p=0.4		
**Retrospective studies**		
First use before age 50	666	1.19 (1.07-1.33)
First use at age 50-59	731	1.01 (0.91-1.11)
First use at age ≥60	139	0.92 (0.74-1.14)
Heterogeneity: p=0.03		
**All studies**		
First use before age 50	1492	1.28 (1.20-1.37)
First use at age 50-59	1801	1.19 (1.12-1.26)
First use at age ≥60	249	1.02 (0.88-1.19)
Heterogeneity: p=0.02		

†Relative risk in current-or-recent versus never users of HT, stratified by study, centre within study, age and body mass index, and adjusted for parity, use of oral contraceptives, age at menopause and hysterectomy.
